# Characterization of Birdwatching Demand Using a Logit Approach: Comparative Analysis of Source Markets (National vs. Foreign)

**DOI:** 10.3390/ani10060965

**Published:** 2020-06-02

**Authors:** Marcelino Sánchez-Rivero, José-Manuel Sánchez-Martín, Mª Cristina Rodríguez Rangel

**Affiliations:** 1 Faculty of Economics, University of Extremadura, 06006 Badajoz, Spain; sanriver@unex.es; 2 Faculty of Business, Finance and Tourism, University of Extremadura, 10071 Cáceres, Spain; jmsanche@unex.es

**Keywords:** tourism demand, birdwatching, logit modelling, probability, Chow test

## Abstract

**Simple Summary:**

Nature tourism, which includes birdwatching, has experienced a significant boom in recent years, becoming a highly attractive tourist typology. The economic benefit it generates for the destinations in which it develops is accompanied by a greater awareness in society about the importance of conserving its natural resources, which is materialized in a greater channeling of investments aimed at ensuring the correct conservation of natural heritage. Therefore, birdwatching has become a highly sustainable tourist product, with which a beneficial interaction between humans and the environment is achieved. In order to achieve these objectives, it is necessary to know exhaustively what the target market is like, that is, the profile of the birdwatcher; what are their sociodemographic characteristics, how do they behave at their destination, or what characteristics influence a greater probability of doing this practice. The purpose of this work is to identify the main characteristics of the demand in a territory that, due to its excellent endowment of natural resources, can become a priority destination for this type of tourism, the Extremadura region (Spain).

**Abstract:**

Birdwatching is a tourism activity that relates closely to protected natural spaces and that helps contribute to the balance between economic, social and environmental aspects of sustainability. In some European countries (the United Kingdom, Germany, Holland), this recreational activity has a large number of followers, making it a new segment of tourist demand with great possibilities for growth. The objective of this study is to identify the main characteristics of the demand for birdwatching in one of the European territories having a high resource supply, as is the case with Extremadura (Spain). To do this, a logit modelization has been proposed in order to estimate the probability of going birdwatching in the region, based on a random sample of over 3000 tourists that visited the region in 2017. This characterization of birdwatching demand was carried out using variables such as gender, age, type of travel, type of lodging, and assessment of tourism services. Given that the national and the foreign demand of this tourism modality may present distinct behaviors, and therefore, specific characterizations, a structural change test (Chow test) was also conducted in order to determine to what extent these two segments of demand, based on the source markets, have (or do not have) distinguishing features.

## 1. Introduction

In a world in which population concentrations in large cities are ever increasing and where pollution and water supply/distribution problems are of growing concern, the search for contact with nature is becoming more and more common. Therefore, in contrast to the more conventional or well-established tourism types (from a market life cycle perspective), such as sun and beach tourism or cultural tourism, some new ways of experiencing tourism are becoming increasingly popular, such as ecotourism. Within this field of ecotourism, which is based on contact with nature, birdwatching is, perhaps, the most rapidly growing of recent years.

Various authors have noted that birdwatching tourism may contribute to local economic growth and help finance the protection and conservation of bird populations [1,2,3]. Other studies have shown that birdwatching tourists spend more money than other tourists, mainly on specialized equipment and services [4,5]. Some authors have even demonstrated the social importance of birdwatching [6].

In contrast to these positive effects of birdwatching, some authors have suggested that there may be negative effects of this tourism practice, mainly of an environmental nature. The negative effects of this practice may include bothering the birds and damaging their natural habitat [7,8,9,10] or the indirect impact of the birdwatching activity on the life cycle of the birds [11]. Other obstacles to the development of this tourism modality include a lack of infrastructures (accesses, hides, watching towers) [12] or marketing strategies that do not attract a sufficient number of bird lovers to ensure that birdwatching truly contributes to local economies [13].

In any case, and despite the strengths and weaknesses of birdwatching as discussed in the literature, it is clear that, in the area of tourism research, studies on birdwatching from a demand perspective, such as that used in this study, are quite insufficient, especially when compared to those considering other more popular tourism modalities. So, the words of Steven et al. [3]: “Research interest in avitourism is still relatively embryonic compared with higher order markets such as nature-based or wildlife tourism” clearly reflect the need to take a closer look at the analysis of the demand for birdwatching, a need that we attempt to at least partially cover in this work. As will be seen in this work, efforts have been made to characterize birders based on their level of motivation, associating them with a series of sociodemographic characteristics and making a special distinction between the origin of the tourist, differentiating between the national and foreign tourist. All this invites us to think that there could be significant differences between the two markets that should be taken into account by the destination managers in order to correctly segment the birdwatching market.

For all these reasons, the objective of this paper is, on the one hand, to characterize the demand for birdwatching in one of the European regions having a high number of resources and a more decided public policy for the development of this sub-typology of ecotourism, Extremadura (Spain), on the other hand, to determine if the sociodemographic characteristics associated with the birders who visit the region may present differences depending on the market of origin. To achieve this objective, the present work divides its analysis into two clearly differentiated parts that are described in the following paragraph.

The first part of this empirical analysis aims to determine if the probability of engaging in birdwatching is determined by socio-demographic factors, characterization of the travel and perception of the destination, using a logistic regression model to do so. The second part of the empirical analysis presented in this study aims to conduct a segmentation of the demand for birdwatching based on the source market (national or foreign) through the introduction of a dummy-like control variable. An adaptation of the well-known structural change test or Chow test for logistic regression models permits confirmation of the existence of significant differences in the probability of engaging in birdwatching based on the source market of the birders, and verifies that certain characteristics condition the probability of practicing this modality by Spaniards, but not by foreigners, and vice versa.

The structure followed by this work to achieve its objectives is as follows. After this introduction, a bibliographic search is carried out to frame the tourist activity to be analyzed, as well as the studies previously carried out on the subject, and then in the following section, describe the existing resources in the region under study, Extremadura (Spain). Section 4 and Section 5 collect the methodology and main results of each of the analyses described in the previous paragraph, respectively. Finally, the article concludes by assessing the implications of the empirical results of these sections, on a tourism management level.

## 2. New Segments of Tourism Demand Related to Nature: Birdwatching

The need to have contact with nature, inherent in those living in the large cities, is the main motivation for the creation and development of a tourism modality having a clear tendency for expansion in various countries: Nature tourism [14]. It is, therefore, an emergence of products related to nature within which ecotourism exists, given their distinctive characteristics, presented as that which best complies with the needs of these areas, being an activity that permits the creation of wealth for the local population while at the same time contributing to the conservation of the environment, thus serving as a sustainable activity.

Ecotourism may be defined as environmentally responsible travel to areas that are highly unaltered, in order to enjoy and appreciate nature while at the same time promoting conservation. It has a low environmental impact and offers socio-economic benefits to the local population [15]. The size of this market, on the other hand, which in 2002, according to the World Tourism Organization (WTO) [16] represented 25% of the national demand, is just one more reason why the destinations wish to include products that may satisfy the needs of this growing demand. Within the area of ecotourism, birdwatching stands out, given that it is the most rapidly-growing product of recent years [5,17,18,19,20].

For its part, ornithological tourism can be defined as recreational activity consisting of the observation and identification of birds in their natural habitat [18,21,22,23] having the objective of approaching nature so as to cover the needs of learning, affiliation, achievement, and/or personal recognition [24].

The beginnings of this activity may be attributed to reverend Gilbert White, who began the change towards a new way of understanding the observation and identification of birds, having a large following, in part, as a reflection of the social changes taking place in society: A rural exodus towards a more urban society within the population has led to people feeling a need to reconnect with nature [25].

The founding in 1989, of the Royal Society for the Protection of Birds (RSPB) served as the necessary highlight for the launch of this tourism activity [26] which, according to the latest statistics, and despite the difficulties in precisely defining the sector, represents an activity that results in some 78 million trips, for a total expense of 60,000 million euros in the visited countries [21].

Considering its origin, it is not unusual that its main source markets on a global level are the United Kingdom, the United States, and Canada. So, there is a potential tourist market of approximately 10 million British [27] and 47 million American birdwatchers in 2011 [4].

Despite these data, it is a market in which there is a limited number of statistics to suitably quantify the size of the same, due to, on the one hand, the lack of specific studies carried out to date [28], and, on the other hand, the difficulty in defining the sector given the high level of heterogeneity existing in its demand [29,30,31,32,33,34].

While it is true that, as noted by Steven et al. [3], research in this area remains at a very early state, especially when compared to other tourism products such as nature tourism. In recent years, it can be verified that there is a growing interest in the area of study that is materialized by the proliferation of a series of academic works aimed at creating knowledge about different facets of this tourist typology.

The greatest efforts are being made, as suggested by Steven et al. [3], in the area of the economic impact generated by this activity for the local population [35,36,37,38,39].

The application of the new technologies to the ornithological market [40,41,42,43], together with the possibilities of the development of new destinations [44,45,46] are the most current topics that have been added to the existing literature.

Likewise, it is not unusual that there have been numerous studies conducted to improve the understanding of the specific characteristics of this market. So, the distinct profiles of the birders have been analyzed from a dual perspective; on the one hand, based on the degree of motivation and level of specialization achieved in this activity, and on the other hand, considering the sociodemographic characteristics of the same.

Motivation for birds, as well as degree of specialization achieved in the activity, as previously mentioned, are differential characteristics, since they affect the level of needs as well as the motivations, the level of involvement, and the economic impact generated in the destination [47].

So, distinct classifications have been made for the existing profiles in the birdwatching market. Jones and Buckley [48] distinguished between generalists or occasional birdwatchers and specialists; Torres [49] added a larger nuance, differentiating between specifically ornithological tourists, generic tourists and those with ornithological references. Then, Jones and Buckley [48] added economic capacity to the distinct motivations and finally, Fernández et al. [28] proposed a classification of white, black, and grey blackbirds.

In all of these classifications, each typology is associated with certain sociodemographic characteristics, destination behaviors, motivations, travel types, and selected lodging types, among others, which are provided by the destinations that wish to form a part of these markets, issues that should be considered in order to ensure a satisfactory experience in such a demanding market niche as birdwatching.

To conclude, the differentiation made by tourists attending to the sociodemographic characteristics, especially those of nationality, are also found to be recurring when it comes to gaining understanding of the functioning of the birdwatching market. So, if we analyze the different works that have been carried out on tourism destinations in Spain, we find that a distinction tends to be made between the national and international tourist profiles [28,50].

Upon analyzing the different profiles, in regard to nationality, changes are observed in distinct aspects, such as age, travel duration, behavior in destination, tourism expenses spent, and other aspects. These changes may be appreciated in the distinct proportions that are obtained upon subdividing the sample when attending to the national tourist variable, but as of this date, the authors are unaware of any further analysis of whether or not this division has statistical foundations. Until now, it was not known if the national and international markets have any differential features. So, in this article, an attempt is made to respond to this question by carrying out a Chow test which permits knowledge of whether or not there are differential features that should be taken into consideration in these markets.

The verification of the possible differences between the national market vs. international has important practical implications since, if significant differences are found between the two markets, this information will be a valuable tool to help destination managers to correctly segment the market, achieving more efficient destination management. To identify these characteristics, information obtained in one of the main birdwatching destinations in Spain, Extremadura, which, given its natural resources, gives this tourism typology ideal development opportunities.

The next section offers a description of the reality of birdwatching in the Extremadura region, offering the reader greater knowledge of the state of this tourism sector, and therefore, a better understanding of our study’s results.

## 3. Birdwatching in Extremadura: Analysis of Supply and Demand

Extremadura, as revealed in the 2004 Nature Tourism Impulse Plan in Spain, has potential to become a top reference destination as it hosts some of the richest ornithological environments of all of Europe, given the huge quantity of emblematic species that live there as well as the ease of their observation.

The maintenance of agrarian forms of traditional production offers a high level of conservation of the typical landscape of this Spanish region, which together with a low population density, have made Extremadura a high quality ecosystem for bird colonies [51].

Nature tourists, especially those going birdwatching, place greater value on the state of conservation of the natural habitat than on the existence of tourism infrastructures [52,53]. In this context, the existence of an environment that is rich in biodiversity and well maintained is appealing to tourists, while also serving as a development opportunity for the rural settings [54]. So, it is not unusual that, along with the growth of the demand for nature tourism, there has also been a generalized increase in the protection of the spaces via distinct legal figures, thus increasing the awareness of the local population with regards to the need for environmental conservation [32].

In the case at hand, the Government of Extremadura has designated 69 Areas of Special Protection for Birds (ZEPA). This network is one of the most extensive of the Iberian Peninsula, representing 26.1% of the territory of the region and having a total extension of 1,089,936 hectares. In addition, 74.1% of the Extremadura territory has been included within the inventory of Important Bird Areas (IBA), being the most extensive area in Europe. Thirty percent of its territory is proposed to be a Site of Community Importance (SCI). All of this offers a good idea of the awareness existing in the region as to the need for conserving its natural resources to ensure its future economic development.

Considering the existing resources, it should be mentioned that the region is not characterized by having a great variety of birds, although it is characterized by the large concentration of the species that are present, and for having a large concentration of birds that are in danger of extinction. Some 35% of the taxa of protected species in Europe are present in this region. This includes, among others, the presence of the Iberian imperial eagle, Bonelli’s eagle, black vulture, black-winged kite, lesser kestrel, black stork, white stork, great bustard, little bustard, pin-tailed sandgrouse, black-bellied sandgrouse, collared pratincole, great spotted cuckoo, red-necked nightjar, roller, European bee-eater, chalk-browed mockingbird, black wheatear, spectacled warbler, Iberian magpie, and common crane [55].

The resources existing in the region have been accompanied by the efforts made to offer the necessary infrastructures for the practicing of this activity, such as the construction of fixed observatories and the habilitation of areas for this purpose [56].

In addition to providing this area with the necessary infrastructures, communication actions are also being carried out to promote, on a national and international level, the richness of the existing resources in the region. In this way, potential tourists are given the information that they need to organize satisfactory birdwatching experiences in the region. The creation of the website www.birdinginextremadura.com, by the Counsel of Economy and Infrastructure of the Government of Extremadura, is a good tool for these purposes. It was created in order to serve as a reference guide for the organization of birdwatching trips to the region, providing information on routes and itineraries, recommended seasons, tourism services as well as other information that is of use for the planning of this practice.

Another example of the efforts being made is the celebration of ornithological fairs, serving as a useful instrument to promote and commercialize. In Extremadura, a series of events is currently being held, including the following: The Bird Festival in Cáceres, the Crane Festival in the Periurban Park of Dehesa Moheda Alta (Navalvillar de Pela), the Week of the Stork in Malpartida de Cáceres, and especially relevant, and the International Fair of Ornithological Tourism (FIO) in the Monfragüe National Park.

The FIO which, in its 2019 edition, celebrated its fourteenth edition, received some 15,600 visitors, according to data provided by the National Park and offered a commodity market in which over 400 meetings were organized. Based on visitor data, the variety of international destinations present and the longevity of the same, it may be considered the most important fair of this sort in this sector in Spain, and the second in all of Europe.

The combination of all of these factors has turned this region into one of the main birdwatching destinations of Spain, with the Parque Nacional de Monfragüe, as the flagship of this segment in Extremadura, being one of the consolidated destinations of the country, along with the Parque Natural de Doñana and the Strait of Gibraltar, and being the second destination in terms of number of tourists received, only behind Andalusia.

As for the data existing on the current demand of birdwatching in Extremadura, it should be noted that, as occurred on a global level, this sector is characterized by a lack of statistics permitting the quantification of the market size.

On the other hand, and due to the importance of this sector in terms of economic development of the region, distinct organisms have made great efforts to permit the understanding of the current reality of this demand. So, in 2011 the local administration requested a study to analyze the demand profile: The Study of Effective Demand of Ornithological Tourism in Extremadura, which, together with the previous study that was commissioned by the General Secretariat of Tourism (SGT) in 2007 [50], revealed some interesting data on the profile of the tourist visiting Extremadura to go birdwatching.

International tourism is quite relevant within the ornithological sector of the region. Some 46.8% of the birders are international tourists, with the main source markets being England, followed by Holland and Germany, representing in each case, a total of 26.3%, 24.9%, and 13.5%, respectively. National tourists come mainly from Madrid, Castilla y León, and Andalusia.

As for the age of the tourists, national tourists have a lower mean age, at 42, a fact which was detected in the study conducted by the SGT in 2007 [52]. This lower mean age may be due to the fact that practicing birdwatching in Spain does not have the same tradition as it does in other countries, and therefore, it is practiced by a younger population.

As for the mean stay, it is seen that this factor is greater in the international case, with national tourists having a mean stay in the region of 4.06 nights, while the international tourists have a mean of 7.06 nights. This, along with the greater mean expenditure made per tourist gives the international market an economic potential that should not be ignored. Almost all of the tourists organized the trip on their own, without significant differences being seen based on nationality.

Regarding the main motivation for the trip, there are some relevant differences found for both markets. So, in over half of the international travelers, bird watching was the main reason for their visit, representing 67% of the cases. On the other hand, the national market has a more balanced motivation, with birdwatching being complementary to other activities and only being found to be the main reason in 45% of the surveyed cases.

Having presented the main conclusions found from the study of the birdwatching sector in Extremadura to date, we should note some of the main limitations of the same. As the authors of the study have mentioned, the difficulties of conducting a study of this sort on such an unknown segment forces them to concentrate their work efforts on very specific geographic areas, the Monfragüe National Park and the Periurban Park of Dehesa Moheda Alta, which limits the possibility of gaining a global view of the sector across the entire region. While it is certain that given the weight of these two destinations on the sector in this region the results obtained may be considered representative, the geographic limitation may introduce certain biases in some of the results that have been obtained.

This study extends the study field to the entire regional territory, using a sample having none of the previously described geographic limitations. In this way, we attempt to determine which of the analyzed factors, within a set of previously selected variables, increase the probability of going birdwatching in the region. To do so, variables have been selected, which, according to the existing bibliography, influence the tourist’s behavior and preference in the destination, such as age, gender, type of travel, and lodging type selected [40]. Then, it shall be determined if the results obtained remain stable when subdividing the sample based on the nationality of the tourist, reflecting the potential structural changes that are identified upon making this division.

## 4. Profile of Birdwatching Demand in Extremadura Using a Logit Approach

### 4.1. Description of Data

The data that will serve to analyze the profile of birdwatching demand in Extremadura have been obtained from the Extremadura Tourism Observatory. More specifically, the survey carried out in the network of tourist offices in the region during 2017 has been used to know the profile of the tourist who visits the region. In addition to including issues related to gender, nationality, age range, people accompanying the tourist on the trip, the type of accommodation chosen for the night or the assessment of various tourist services in the area visited, one of the questions of the survey referred to the type of tourist activity carried out during the visit, one of them being birdwatching. In this way, and after carrying out a purification process of the original database, the final sample of 3634 tourists was obtained, which is the one that has been used to estimate the logit model. Of this final sample, 3221 surveys correspond to national tourists (88% of the total) and 413 surveys correspond to foreign tourists (12% of the total), a distribution that corresponds approximately to the distribution by nationality of the global tourism demand of Extremadura over the years (85% domestic tourists; 15% foreign tourists).

Additionally, the practice (or not) of birdwatching in the region has a binary nature, depending on whether the tourist has indicated (or not) the option of practicing this activity during their stay in Extremadura. Finally, the categorical nature of the variables included in the survey that can facilitate the definition of a profile of the birdwatching practitioner (nationality, type of accommodation, type of travel, age segment, etc.) requires the use of dichotomous explanatory variables (as many for each analyzed characteristic as categories considered in the survey minus one). Other variables (those related to the assessment of tourist services) can be considered as scale variables and have been introduced as such in the statistical model detailed below as explanatory variables of the probability of practicing birdwatching.

### 4.2. Methodology

To expand upon the profile analysis of the demand for birdwatching, a regression analysis has been used in which the dependent variable ($Y_{i}$) is a dummy variable, which will have the value of 1 if the tourist has gone birdwatching during his/her visit to Extremadura, and the value 0 in the opposite case. Given the binary nature of this dependent variable, the following binary logistic regression model (or logit model) has been proposed:

(1) $$P\left(Y_{i}=1\right)=\frac{\exp\left(z\right)}{1+\exp\left(z\right)}$$

with

(2) $$z=\beta_{0}+\beta_{1}\;GEN_{i}+\beta_{2}\;AG1_{i}+\beta_{3}\;AG2_{i}+\beta_{4}\;COMP1_{i}+\beta_{5}\;COMP2_{i}+\;\beta_{6}\;H1_{i}+\beta_{7}\;H2_{i}+\beta_{8}\;H3_{i}+\beta_{9}\;VAL\_ALOJ_{i}+\beta_{10}\;VAL\_REST_{i}+\beta_{11}\;VAL\_EMP_{i}+\;\beta_{12}\;VAL\_NAT_{i}.$$

In which $P\left(Y_{i}=1\right)$ represents the probability that the tourist i goes birdwatching in Extremadura, and where the explanatory variables of the model may be grouped in three main categories: Sociodemographic variables: GEN: gender (1 = male; 0 = female); AG1: age (1 = 35 years or less; 0 = others); AG2: age (1 = between 35 and 55 years of age; 0 = others). Note: Over 55 years of age (AG1 = AG2 = 0). Variables of trip characterization: COMP1: type of travel (1 = in couple or in family; 0 = others); COMP2: type of travel (1 = with friends or in group; 0 = others). Note: Alone (COMP1 = COMP2 = 0); H1: type of lodging selected for overnight stay (1 = hotel; 0 = others); H2: type of lodging selected for overnight stay (1 = rural lodging; 0 = others); H3: type of lodging selected for overnight stay (1 = apartment, camping or hostel; 0 = others). Note: other lodgings (H1 = H2 = H3 = 0). Variables of assessment of destination: VAL_ALOJ: assessment, on a scale of 0 to 10 points, of the lodging offer; VAL_REST: assessment, on a scale of 0 to 10 points, of the restaurant offer; VAL_EMP: assessment, on a scale of 0 to 10 points, of the tourism activity company; VAL_NAT: assessment, on a scale of 0 to 10 points, of the conservation of the natural heritage.

The logit model [57,58,59,60] has been frequently used in the tourism research field. So, for example, it has been used for issues as diverse as the identification of the determinant factors of innovation in tourism [61,62], establishing space–time relations between hotels in urban tourism destinations [63], determining the influence of the High Speed Rail on the probability of returning to visit a destination [64], studying the consumption of local food in rural tourism [65], analyzing the behavior of the tourist in the consumption of certain products [66], analyzing air quality inside museums [67], or determining the predictive factors of the tourist’s loyalty to a destination [68]. It is, therefore, a widely contrasted methodology in the field of tourism research.

### 4.3. Results

The results of the model estimation (1) using the Gretl statistics package are shown in Table 1. In this table, we can see that no sociodemographic variable is statistically significant in said model. On the other hand, the explanatory variables related to the characterization of the trip do in fact contribute, in general, to explaining the odds ratio between the probability of going birdwatching in Extremadura and not doing so. Specifically, the lodging type selected by the tourist to stay in while visiting the region determines a greater probability of going birdwatching. So, the interpretation of the exp (β), in the case of ceteris paribus, allowed us to affirm that tourists lodging in apartments, camping sites, or hostels had a greater probability of engaging in birdwatching as compared to the other tourists. Those tourists staying in rural lodgings also revealed higher probabilities of going birdwatching, although with a slightly lower odds ratio (1.565). Those staying in hotels also had a higher propensity to go birdwatching, although it is lower than that estimated for apartments, camping sites or hostels or that estimated for stays in rural lodgings. Therefore, the model estimation (1) allows us to determine that the tourists that were most likely to engage in birdwatching tended to prefer extra-hotel establishments for their overnight stays, which typically are less expensive (especially the hostel and camping sites and to a lesser extent, the rural lodgings) than the hotel establishments. Although other factors could also be influencing the greater predilection for this type of accommodation, such as a more appropriate location, as they are usually closer to the typical places of practice of this activity.

It has also been determined, based on the model estimation (1) that there is a greater predisposition to engage in birdwatching in tourists that travel with friends or in groups. Therefore, another differentiating characteristic of the birdwatching demand is its greater inclination to travel in groups or in the company of friends, instead of doing so accompanied by a partner or family or alone.

However, the act of considering the demand for birdwatching in an aggregate manner, without differentiating between national and foreign tourists, may be hiding some statistically significant relationships between some explanatory variables and the probability of going birdwatching. In fact, in the measure in which the behavior of the national tourist may differ from that of the foreign tourist, it is possible that variables that have not proven to be significant in the model (1) are in fact significant in one of these two segments of demand (defined according to origin), or vice versa.

Therefore, it is necessary to introduce the origin (national or foreign) of the analyzed tourists as a control variable in the model (1) in order to determine if the segmentation of the birdwatching demand in the function of the origin of national markets or foreign markets results in differentiated behaviors.

## 5. Segmentation of Demand Based on Source Markets: Analysis of Structural Change Using the Chow Test

### 5.1. Methodology

The test for structural change known as the Chow test [69], is typically used with conventional regression models to determine if upon dividing a model into two subsamples, stability exists in the model parameters. In a conventional regression model, this Chow test includes an F statistic in which the sum of squares of the errors of the model estimated with the total sample (restricted model) are compared with the sum of squares of the errors of the models estimated based on each subsample (non-restricted model).

But when the estimated regression model is a binary logistic regression model, as in this case, this Chow test is conducted like a likelihood ratio test between the restricted (pooled) logit model (model (1)) and the non-restricted logit model. This last model defines the z function as follows:

(3) $$z=\beta_{0}+\beta_{1}\;GEN_{i}+\beta_{2}\;AG1_{i}+\beta_{3}\;AG2_{i}+\beta_{4}\;COMP1_{i}+\beta_{5}\;COMP2_{i}+\;\beta_{6}\;H1_{i}+\beta_{7}\;H2_{i}+\beta_{8}\;H3_{i}+\beta_{9}\;VAL\_ALOJ_{i}+\beta_{10}\;VAL\_REST_{i}+\beta_{11}\;VAL\_EMP_{i}+\;\beta_{12}\;VAL\_NAT_{i}+\beta_{13}\;D_{i}+\beta_{14}\;GEN_{i}\times D_{i}+\beta_{15}\;AG1_{i}\times D_{i}+\beta_{16}\;AG2_{i}\times D_{i}+\;\beta_{17}\;COMP1_{i}\times D_{i}+\beta_{18}\;COMP2_{i}\times D_{i}+\beta_{19}\;H1_{i}\times D_{i}+\beta_{20}\;H2_{i}\times D_{i}+\beta_{21}\;H3_{i}\times D_{i}+\;\beta_{22}\;VAL\_ALOJ_{i}\times D_{i}+\beta_{23}\;VAL\_REST_{i}\times D_{i}+\beta_{24}\;VAL\_EMP_{i}\times D_{i}+\;\beta_{25}\;VAL\_NAT_{i}\times D_{i}$$

where the $D_{i}$ variable is a control variable, taking on the value of 1 if the tourist is a national and 0 if the tourist is a foreigner.

In our case, and given the fact that the output of the Gretl results offers the logarithm of the Log-likelihood function, the contrast that has been used is the log-likelihood ratio test between both models, as shown in the following expression:

(4) $$D=-2\left[\log\left(\Lambda_{1}\right)-\log\left(\Lambda_{2}\right)\right]$$

where $\log\left(\Lambda_{1}\right)$ is the logarithm of the log-likelihood function of the restricted model (model (1)) and $\log\left(\Lambda_{2}\right)$ is the logarithm of the log-likelihood function of the non-restricted model (model (2)).

Wilk [70] demonstrates that the D statistic follows a asymptotic $\chi^{2}$ distribution with df1−df2 degrees of liberty, where df1 and df2 represent, respectively, the degrees of liberty of the models (1) and (2). If the p-value associated with this D statistic is lower than the level of significance, the presence of a structural change may be admitted and therefore, it would be possible to conclude that significant differences exist in the adjustment of the binary logit model for the national and foreign tourists.

Although this test for conventional structure change (that is, that is based on a classic regression model) has also been used quite frequently in tourism research [71,72,73,74,75], its use with logistic regression models and therefore, its contrast through a likelihood ratio test, is practically non-existent in the field of tourism research. Thus, this work presents a methodological novelty in the field of tourism research.

### 5.2. Results

The estimations of this model (2) are presented in Table 2. In said table, we can see that some of the parameters of the interaction between the explanatory variables of the model and the control variable $D_{i}$ are statistically significant, leading us to suspect that a structural change exists and that, therefore, there may be differences in the logit models estimated for the segment of national tourists and foreign tourists, respectively.

The D statistic in this case had an empirical value of 135.318 and followed an asymptotic $\chi^{2}$ distribution with 13 degrees of freedom, and with an associated *p*-value of 0.000. Therefore, it is possible to conclude that the control variable $D_{i}$ is responsible for a structural change in the model (1) and that, therefore, the estimation of the probability of going birdwatching is determined by distinct explanatory variables (or by the same ones, but with unequal influence) for the segment of national tourists and for that of foreign tourists.

In light of this result, a new model (1) has been estimated for the two subsamples of national tourists and foreign tourists. The results of these estimations are shown in Table 3 and Table 4, respectively.

In the case of the national tourists, the only variables that determine a greater or lower probability of going birdwatching are those related to lodging. So, the variables H1, H2, and H3 are statistically significant at 5%, with the β coefficients being estimated as positive and, therefore with Exp (β) values that are greater than 1. So, the greatest probability of going birdwatching for the national tourists is found for those that lodge in apartments, camping sites or hostels (0.647; 1.911), followed by those staying in rural lodgings (0.460; 1.584) and those staying in hotels (0.280; 1.323).

In the case of foreign tourists, a greater number of variables can determine a higher or lower probability of going birdwatching in the region. As observed in Table 4, the variables AG1 and AG2 are statistically significant at 5%. The β coefficients of both variables are negative, so the Exp(β) values are lower than 1. Therefore, we see that in this tourist segment, those who are between the ages of 35 and 55 (−0.406; 0.666), but, especially, the younger ones (35 or below) (−1.577; 0.207) had a lower probability of engaging in birdwatching than the older tourists (over the age of 55). The H2 variable is also statistically significant at 5%, therefore it may be concluded that the foreign tourists who stayed in rural lodgings had a considerably greater probability of practicing birdwatching than those that stayed in other lodging types (1.090; 2.975). Finally, the VAL_NAT variable is also statistically significant at 10%, such that the positive β coefficient indicates that the greater the mean assessment made by the foreign tourists for the conservation of the region’s natural heritage, the greater the probability that these tourists go birdwatching in this same region.

**Figure 1:**
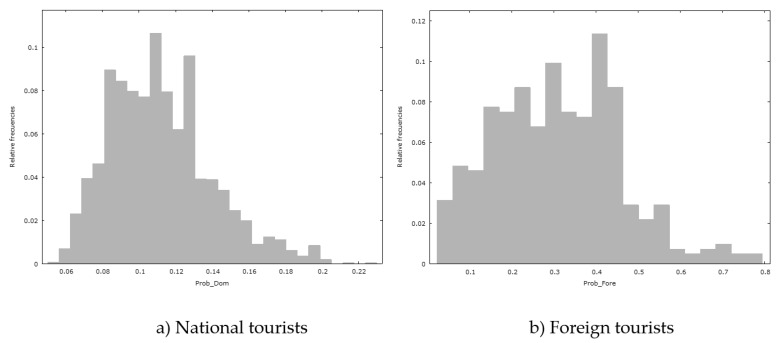
Frequency histogram of the estimated probabilities of going birdwatching: (**a**) Histogram for national tourists; (**b**) Histogram for foreign tourists.

Having estimated and interpreted the binary logistic regression models for the national tourists and foreign tourists, the estimated probabilities have been obtained, according to these models, for going birdwatching in Extremadura. The relative frequency histogram of these estimated probabilities is shown in Figure 1 for both (a) national and (b) foreign tourists. In this figure, the X axis represents the estimated probabilities for going birdwatching in Extremadura, and the Y axis represents the relative frequencies for these probabilities. Both these histograms as well as the fact that the mean estimated probability reaches 11.18% (s.d.: 0.0278) for national tourists and 30.99% (s.d.: 0.1494) for foreign tourists, makes us suspect that there are statistically significant differences in the mean values of these estimated probabilities.

Therefore, a t-test was conducted in which the groups of tourists have been defined based on their nationality (Group 1: nationals; Group 2: foreign). This t test has been carried out for each of the different categories of sociodemographic variables and for the characterization of the travel as considered in this study. The results of these t tests are presented in Table 5.

As we can see, the difference between the mean probabilities of going birdwatching for the national tourists and for the foreign tourists was statistically significant in all cases, with the only exception being the tourists who were a maximum of 35 years of age. In fact, for the youngest tourists, the probability of observing birds during their stay in Extremadura was approximately 12%, both for the national tourists as well as for the foreign tourists. For tourists 35 years of age and older, the differences in probabilities were considerably greater for the foreign tourists, with this difference increasing as the age of the tourist increases. So, for those over the age of 55, a mean probability of engaging in birdwatching in Extremadura has been calculated at 41.46% for foreign tourists and only 9.72% for national tourists.

Considerable differences also exist between the national and foreign tourists with reference to gender. Just like there were no major differences between national men and women (between 10% and 12% probability) and between foreign men and women (between 30% and 32%), it was confirmed that foreign tourists, both men and women, had a mean probability of practicing birdwatching that exceeded that of the national tourists by some 20 percentage points.

Substantial differences were also detected between national and foreign tourists when they travelled in couples or in family. While the national tourists had a mean probability of 11.5%, the foreign tourists had a probability of going birdwatching of over 38%. When the tourists travelled with a friend or in a group, or when they travelled on their own, differences in probability were also recorded in favor of the foreign tourists, although this case is not as evident as when they travelled in the company of their family or as a couple.

Finally, there was also a clear predisposition of the ornithological tourists for certain types of lodging. Specifically, foreign tourists that stayed in rural lodgings had a probability of over 50% of going birdwatching, as compared to less than 13% of the national tourists who spent the night in this type of lodging. There was also a major difference between national and foreign tourists when spending the night in hotels, since the former had an estimated probability of going birdwatching of 11%, while the second had a mean probability of almost 33%. Finally, foreigners who stayed in apartments, camping sites, hostels, or other types of establishments had a 26% probability of going birdwatching, over 11 more percentage points than Spaniards who spent the night in apartments, camping sites, or hostels and 17 percentage points more than Spaniards who stayed in other types of establishments.

## 6. Conclusions

The tourism market is becoming ever more specialized, creating market niches that are more and more specific in order to satisfy the more demanding tourist who seeks an authentic experience from his/her travels and wishes to enjoy the natural environment as an escape from the everyday routine.

Birdwatching, as a market niche, stands out given its strong growth rate within the area of ecotourism, presenting certain destinations as potential engines of development. This is especially the case for rural and less developed destinations which have a higher degree of conservation of their natural resources.

Managers of these destinations are aware of the benefits, both economic and social, that this tourism segment may offer them, given both the economic impact that would help to complement the agrarian activities, and the increased awareness of the care for natural resources, creating an economic development model in which sustainability serves as the cornerstone.

So the interest that birdwatching arouses among some of these territories is hardly surprising, as they wish to form a part of this market, and therefore are carrying out actions that will help position them optimally as a birder destination. These actions include the creation of the infrastructures necessary to observe birds, the cataloging of bird species, and tourism promotion and product development.

Until now, the current literature has demonstrated the heterogeneity of the demand of this sector. So, efforts made to detect and characterize the distinct tourist profiles are essential in order to have a proper knowledge of the market. Based on sociodemographic characteristics, the type of travel, or motivation of the tourist, and their behavior in the destination will differ. Therefore, it is necessary to have a proper characterization of the potential tourists in order to provide the destination with the necessary infrastructure and to ensure that the experience here is of the desired quality.

For the section of tourism promotion of a regional government that aspires to convert birdwatching into a significant tourism offering on a national and international level, knowledge of the characteristics of the demand segments based on the source market of origin (national or foreign) is essential in order to offer a suitable promotion policy.

The results obtained show that for a correct management of the birdwatching market, it is essential to segment the market according to the origin of the tourist, national vs. foreign, since the characteristics of each of the profiles is determined by this aspect.

While confirming that for birders in general, and for the particular case of national birders, only the type of accommodation chosen can influence a greater probability of practicing birdwatching when analyzing the foreign tourist, it is observed that it is tourists over 55 years of age who mainly stay in rural accommodations, and a higher average valuation is given to the state of conservation of the environment by those who have a greater probability of practicing this tourist typology when they visit the region. Destination managers should take these characteristics into account when designing destination planning strategies.

In addition, it has been found that foreign tourists who are more likely to practice birdwatching are those who give a higher average valuation to the state of conservation of natural heritage. Therefore, it is evident that for the sustainability of this sector it is necessary to increase awareness of the importance of the natural environment, since only in this way will the region be able to position itself as a benchmark in this type of tourism. Destination managers must be aware of this reality, making investments that are necessary for its direct conservation, as well as to increase the awareness of society and involve it in this work; only in this way will sustainable management of birdwatching be achieved in the region. The fact that the probability of a foreign tourist engaging in birdwatching in the region is much higher than the probability of a national tourist doing so is quite significant in various respects. So, it is necessary for promotional campaigns to be directed at foreigners (especially central Europeans in this case) and for international fairs to be presented, both general ones (World Travel Market, ITB, BTL, etc.) as well as more specific ones (Birdfair, Dutch Bird Fair, Gouden Ring Show, etc.). The promotional campaigns celebrated in Spain’s tourism fairs (such as FITUR, INTUR, FIO, etc.) are less effective. Furthermore, it is necessary to have good communication to facilitate the access of these foreign tourists from the closest airports (Madrid and Lisbon) to the region, making it necessary to design a transport strategy for intermodal passengers, given the deficient air and railway transport of this region.

On the other hand, the fact that the tourists with the greatest probability of practicing birdwatching in the region are foreigners over the age of 55 demands a complementary tourism offer design that is specifically directed at this age segment. While birdwatching will be the main motivation for these tourists, a good gastronomical offering along with a quality cultural and historic offer and the signing of collaboration agreements between ornithology companies and the regional spas may complement the visits of these tourists, while also extending their stays in the region, with the resulting effects on the regional tourism gross domestic product (GDP), (foreign tourists, in general, have higher mean tourism expenditures than national tourists).

Finally, given that the probability that a foreign tourist spending the night in a rural lodging will go birdwatching is over 50%, it is clear that the owners of these lodgings should have a greater knowledge of foreign languages than they currently do. Thus, the fluidity of speaking English and knowing some German or Dutch (the three main European source countries for birdwatching) are basic issues when attempting to offer quality tourism services to these tourists.

Based on that which has been presented in this work, the importance of the national tourist profile, versus that of the international tourist, is evident, given the differences existing between both markets. This should be taken into consideration when creating a development strategy to follow.

Finally, although there is no previous study to the authors’ knowledge that allows the results obtained in the analyzed territory to be compared, it is possible to take as a background the study carried out by the General Sub-directorate for Tourism Quality and Innovation [50] which obtained similar results, although it limited its study framework to certain strategic enclaves for the practice of this activity in the region. Thus, while for some sociodemographic characteristics that influence the greater probability of practicing birdwatching, such as age, sex, or form of travel, similar conclusions are reached, the same does not occur when analyzing the type of majority accommodation in the case of a national tourist. Thus, while the analyzed study shows a greater preference for rural accommodation for both birder profiles, this study only confirms this trend for the case of foreign birders, with the national birder having a greater preference for accommodation in hostels or campsites. This difference may be based on the difference in the territorial framework analyzed, since it could happen that when considering only certain strategic enclaves as reference, they do not have a complete offer, as is to be expected if the entire regional territory is considered.

One future research line may propose the expansion of the geographic area of study to include other regions, in order to verify if the results obtained are characteristic of the Extremadura birdwatching market, or if, to the contrary, they may be extended to the entire national market. Likewise, it would be interesting to analyze how the geographical aspects, such as the distance traveled, influence the profile and behavior of the tourist at destination.

## Figures and Tables

**Table 1:** Estimation of the binary logistic regression model (1).

Explanatory Variables	β	S.E.	z	Wald	*p*-Value	Sig. ^a^	Exp (β)
GEN	0.101	0.099	1.016	1.032	0.310		1.106
AG1	−0.215	0.154	−1.390	1.932	0.165		0.807
AG2	−0.075	0.118	−0.634	0.402	0.526		0.928
COMP1	−0.052	0.181	−0.286	0.082	0.775		0.950
COMP2	−0.404	0.210	−1.922	3.694	0.055	*	0.668
H1	0.294	0.124	2.362	5.579	0.018	**	1.341
H2	0.448	0.162	2.766	7.651	0.006	***	1.565
H3	0.636	0.159	4.005	16.040	<0.001	***	1.890
VAL_ALOJ	−0.086	0.049	−1.749	3.059	0.080	*	0.918
VAL_REST	0.074	0.062	1.193	1.423	0.233		1.077
VAL_EMP	−0.017	0.040	−0.419	0.176	0.675		0.984
VAL_NAT	0.055	0.052	1.051	1.105	0.293		1.056
Constant	−2.253	0.464	−4.853	23.552	<0.001	***	0.105

Log-likelihood: −1416.419; Schwarz criterion: 2939.413; Akaike criterion: 2858.838; Hannan–Quinn criterion: 2887.540; McFadden’s R^2^: 0.012; Number of cases correctly predicted: 3146 (86.6%); Ratio likelihood test: Chi-Square (12 df) = 34.0692 (*p*-value: <0.001) ^a^: * significant at 10% level; ** significant at 5% level; *** significant at 1% level; Source: own elaboration.

**Table 2:** Estimation of the binary logistic regression model (2). Non-restricted model.

Explanatory Variables.	β	S.E.	z	Wald	*p*-Value	Sig. ^a^	Exp (β)
GEN	0.074	0.230	0.323	0.104	0.747		1.077
AG1	−1.577	0.457	3.454	11.929	0.001	***	0.207
AG2	−0.406	0.251	−1.619	3.825	0.105		0.666
COMP1	0.714	0.350	2.041	4.166	0.041	**	2.042
COMP2	−0.194	0.433	−0.447	0.200	0.655		0.824
H1	−0.006	0.296	−0.020	0.000	0.984		0.994
H2	1.090	0.467	2.332	5.440	0.020	**	2.975
H3	0.144	0.354	0.408	0.166	0.684		1.155
VAL_ALOJ	−0.255	0.159	−1.601	2.563	0.109		0.775
VAL_REST	0.092	0.169	0.546	0.298	0.585		1.097
VAL_EMP	−0.065	0.122	−0.529	0.003	0.597		0.994
VAL_NAT	0.232	0.127	1.821	1.709	0.069	*	1.261
D	−1.377	1.262	−1.092	1.191	0.275		0.252
GEN × D	0.106	0.257	0.411	0.169	0.681		1.111
AG1 × D	1.784	0.489	3.650	13.319	<0.001	***	5.953
AG2 × D	0.549	0.288	1.906	3.632	0.057	*	1.732
COMP1 × D	−0.851	0.410	−2.074	4.301	0.038	**	0.427
COMP2 × D	−0.196	0.498	−0.394	0.155	0.694		0.822
H1 × D	0.286	0.329	0.869	0.755	0.385		1.331
H2 × D	−0.630	0.500	−1.260	1.586	0.208		0.533
H3 × D	0.503	0.398	1.265	1.599	0.206		1.654
VAL_ALOJ × D	0.241	0.169	1.426	2.035	0.154		1.272
VAL_REST × D	−0.027	0.183	−0.147	0.022	0.883		0.973
VAL_EMP × D	0.0633	0.130	0.489	0.239	0.625		1.065
VAL_NAT × D	−0.267	0.140	−1.906	3.632	0.057	*	0.766
Constant	−1.135	1.139	−0.997	0.993	0.319		0.321

Log-likelihood: −1348.760; Schwarz criterion: 2910.671; Akaike criterion: 2749.520; Hannan-Quinn criterion: 2806.923; McFadden’s R^2^: 0.059; Number of cases correctly predicted: 3161 (87.0%); Ratio likelihood test: Chi-Square (25 df) = 169.387 (*p*-value: 0.000); ^a^: * significant at 10% level; ** significant at 5% level; *** significant at 1% level. Source: own elaboration.

**Table 3:** Estimation of the binary logistic regression model (1) for national tourists.

Explanatory Variables	β	S.E.	z	Wald	*p*-Value	Sig. ^a^	Exp (β)
GEN	0.180	0.114	1.574	2.478	0.116		1.197
AG1	0.207	0.175	1.186	1.406	0.236		1.230
AG2	0.143	0.142	1.008	1.016	0.314		1.154
COMP1	−0.137	0.214	−0.638	0.407	0.523		0.872
COMP2	−0.390	0.245	−1.589	2.525	0.112		0.677
H1	0.280	0.142	1.964	3.859	0.049	**	1.323
H2	0.460	0.178	2.578	6.647	0.001	***	1.584
H3	0.647	0.182	3.553	12.622	<0.001	***	1.911
VAL_ALOJ	−0.015	0.055	−0.263	0.069	0.793		0.986
VAL_REST	0.065	0.070	0.939	0.882	0.348		1.068
VAL_EMP	−0.001	0.044	−0.025	0.001	0.980		0.999
VAL_NAT	−0.035	0.058	−0.601	0.361	0.548		0.966
Constant	−2.512	0.542	−4.632	21.548	<0.001	***	0.081

Sample size: 3221; Log-likelihood: −1115.760; Schwarz criterion: 2336.527; Akaike criterion: 2257.520; Hannan-Quinn criterion: 2285.836; McFadden’s R^2^: 0.011; Number of cases correctly predicted: 2861 (88.8%); Ratio likelihood test: Chi-Square (12 df) = 24.4224 (*p*-value: 0.018) ^a^: * significant at 10% level; ** significant at 5% level; *** significant at 1% level. Source: own elaboration.

**Table 4:** Estimation of the binary logistic regression model (1) for foreign tourists.

Explanatory Variables	β	S.E.	z	Wald	*p*-Value	Sig. ^a^	Exp (β)
GEN	0.074	0.230	0.323	0.104	0.747		1.077
AG1	−1.577	0.457	−3.454	11.929	0.001	***	0.207
AG2	-0.406	0.201	−2.023	4.092	0.022	**	0.666
COMP1	0.714	0.350	2.041	4.166	0.041	**	2.042
COMP2	−0.194	0.433	−0.447	0.200	0.655		0.824
H1	−0.006	0.296	−0.020	0.000	0.984		0.994
H2	1.090	0.467	2.332	5.440	0.020	**	2.975
H3	0.144	0.354	0.408	0.166	0.684		1.155
VAL_ALOJ	−0.255	0.159	−1.601	2.563	0.109		0.775
VAL_REST	0.092	0.169	0.546	0.298	0.585		1.097
VAL_EMP	−0.065	0.122	−0.529	0.279	0.597		0.938
VAL_NAT	0.232	0.127	1.821	3.317	0.069	*	1.261
Constant	−1.135	1.139	−0.997	0.993	0.319		0.321

Sample size: 413; Log-likelihood: −233.0003; Schwarz criterion: 544.3053; Akaike criterion: 492.0005; Hannan-Quinn criterion: 512.6877; McFadden’s R^2^: 0.089; Number of correctly predicted cases: 300 (72.6%); Ratio likelihood test: Chi-Square (12 df) = 45.3286 (*p*-value: <0.001); ^a^: * significant at 10% level; ** significant at 5% level; *** significant at 1% level. Source: own elaboration.

**Table 5:** T-tests of equality of estimated probabilities of going birdwatching for national tourists and foreign tourists.

Explanatory Variables	Average Domestic	Average Foreign	Difference	t Statistics	*p*-Value
Is a man	0.1212	0.3224	−0.2012	−17.46	<0.001
Is a woman	0.1025	0.3000	−0.1975	−20.77	<0.001
Is 35 or younger	0.1163	0.1212	−0.0049	−0.50	0.617
Is between 35 and 55	0.1164	0.3080	−0.1916	−25.69	<0.001
Is over the age of 55	0.0972	0.4146	−0.3174	−25.38	<0.001
Travel in couple or in family	0.1149	0.3825	−0.2676	−32.78	<0.001
Travel with friends or in a group	0.0909	0.1868	−0.0959	−8.97	<0.001
Travel alone	0.1385	0.2113	−0.0728	−5.97	<0.001
Lodge in hotels	0.1106	0.3298	−0.2192	−24.91	<0.001
Stay in rural lodgings	0.1289	0.5185	−0.3896	−12.87	<0.001
Stay in apartments, camping sites or hostels	0.1566	0.2653	−0.1087	−6.73	<0.001
Lodge in other types of establishments	0.0873	0.2600	−0.1727	−40.01	<0.001

Source: own elaboration using SPSS software (IBM, Armonk, NY, USA).
